# Corrosion and Heat Treatment Study of Electroless NiP-Ti Nanocomposite Coatings Deposited on HSLA Steel

**DOI:** 10.3390/nano10101932

**Published:** 2020-09-27

**Authors:** Khuram Shahzad, Eman M. Fayyad, Muddasir Nawaz, Osama Fayyaz, R. A. Shakoor, Mohammad K. Hassan, Malik Adeel Umer, M. N. Baig, A. Raza, Aboubakr M. Abdullah

**Affiliations:** 1 Center for Advanced Materials (CAM), Qatar University, Doha 2713, Qatar; Khuram.phd@scme.nust.edu.pk (K.S.); emfayad@qu.edu.qa (E.M.F.); m.nawaz@qu.edu.qa (M.N.); of1806234@student.qu.edu.qa (O.F.); mohamed.hassan@qu.edu.qa (M.K.H.); bakr@qu.edu.qa (A.M.A.); 2 Department of Materials Engineering, School of Chemical and Materials Engineering, National University of Science and Technology (NUST), Islamabad 44000, Pakistan; 3 Industrial Technology Department, National Center for Physics, Quaid-i-Azam University, Islamabad 2141, Pakistan; mnbaig8167@gmail.com (M.N.B.); aamiraza1@gmail.com (A.R.)

**Keywords:** electroless deposition, Ti-nanoparticles (TNPs), composite coatings, hardness, EIS, oxidation

## Abstract

Corrosion and heat treatment studies are essential to predict the performance and sustainability of the coatings in harsh environments, such as the oil and gas industries. In this study, nickel phosphorus (NiP)–titanium (Ti) nanocomposite coatings (NiP-Ti nanoparticles (TNPs)), containing various concentrations of Ti nanoparticles (TNPs) were deposited on high strength low alloy (HSLA) steel through electroless deposition processing. The concentrations of 0.25, 0.50 and 1.0 g/L TNPs were dispersed in the electroless bath, to obtain NiP-TNPs nanocomposite coatings comprising different Ti contents. Further, the effect of TNPs on the structural, mechanical, corrosion, and heat treatment performance of NiP coatings was thoroughly studied to illustrate the role of TNPs into the NiP matrix. Field emission scanning electron microscope (FESEM) and energy dispersive spectroscopy (EDX) results confirm the successful incorporation of TNPs into the NiP matrix. A substantial improvement in the mechanical response of the NiP matrix was noticed with an increasing amount of TNPs, which reached to its ultimate values (hardness 675 Hv, modulus of elasticity 18.26 GPa, and stiffness 9.02 kN/m) at NiP-0.5TNPs coatings composition. Likewise, the electrochemical impedance spectroscopy measurements confirmed a tremendous increase in the corrosion inhibition efficiency of the NiP coatings with an increasing amount of TNPs, reaching ~96.4% at a composition of NiP-0.5TNPs. In addition, the NiP-TNPs nanocomposite coatings also unveiled better performance after heat treatment than NiP coatings, due to the presence of TNPs into the NiP matrix and the formation of more stable (heat resistant) phases, such as Ni_3_P, Ni_3_Ti, NiO, etc., during the subsequent processing.

## 1. Introduction

Nickel phosphorus (NiP) coatings have propitious mechanical (better hardness, and improved wear and abrasion resistance) and anticorrosion properties, so they have applications in industries like automotive, oil and gas, electronics, and aerospace [1,2,3]. The electroless deposition method is extensively used for the fabrication of NiP coatings owing to its advantages, such as the formation of uniform thickness over the intricate shapes, the homogeneous composition throughout the structure, and the better adhesion with the substrate [4,5,6].

Further, to enhance the properties of electroless NiP coatings, incorporation of metallic or ceramic (micron or nano) particles into the NiP matrix has been carried out, leading to the development of composite coatings. For example, the inclusion of C_3_N_4_, TiN, BN, Al_2_O_3_, TiO_2_, SiO_2_, SiC, B_4_C [7,8,9,10,11,12,13,14], as well as metals like Al, Ti, Cu [15,16,17] can be found in the literature. The insertion of the ceramic particles like Al_2_O_3_ [10] and TiO_2_ [11] into the NiP matrix can ameliorate the hardness and the wear resistance of the NiP coatings. Whereas others, like CeO_2_ and Si_3_N_4_ can increase the corrosion resistance properties of NiP coatings [18,19]. Most of the studies are focused on the addition of ceramic particulates into the NiP matrix, which may have problems like interdiffusion with the ceramic metal interface, and low toughness due to their hard nature [20,21]. In contrast, a metal powder, which exhibits better interface with NiP matrix and has moderate toughness, along with higher hardness and wear resistance, can be used as acceptable reinforcement particles. Titanium (Ti) is a transition metal that has low density, comprises of high strength, thermally stable at high temperature, and demonstrates better corrosion resistance [22]. Therefore, the addition of Ti nanoparticles (TNPs) into to the NiP coatings is a possible way to improve its hardness, toughness, and corrosion resistance. Studies on mechanical, corrosion, and erosion characteristics of electroplated NiP coatings containing Ti particles exist more in the literature [15,23,24,25,26]. However, a few studies are available for electroless NiP-Ti composite coatings describing their mechanical, corrosion, and erosion behavior [27]. Likewise, there are no studies reported on the heat treatment behavior of electroless NiP-Ti nanocomposite (NiP-TNPs) coatings so far. Moreover, electroless deposition of NiP-TNPs nanocomposite coatings on high strength low alloy (HSLA) steel, which is an essential alloy for the aerospace industry, is also not available in the literature. It is pertinent to mention here that like nickel-plated aerospace-grade materials like HSLA steel is also required to face extreme mechanical, thermal, and corrosive conditions during their service [28,29]. Therefore, HSLA steel requires dedicated research to improve their surface properties by applying corrosion and heat resistant coatings. 

In the present study, NiP-TNPs composite coatings were developed by reinforcing the NiP matrix with various concentrations (0.0, 0.25, 0.5, and 1.0 g/L) of Ti nanoparticles (TNPs) through the electroless deposition process. HSLA steel was chosen as a substrate to target its application for the aerospace industry. Further, the effect of TNPs concentration on structural, mechanical, corrosion, and heat treatment behavior of NiP coatings was examined to clarify the superior performance of these nanocomposite coating systems. The coating morphology and chemical composition were studied using field emission scanning electron microscope (FESEM), EDX, and X-ray diffraction (XRD) techniques. The mechanical evaluation of the developed coatings was performed through the microhardness and nanoindentation techniques. The anticorrosion response of the developed coatings was assessed through the electrochemical impedance spectroscopy (EIS) and potentiodynamic polarization techniques in 3.5 wt. % NaCl solutions.

## 2. Materials and Method

### 2.1. Substrates and Coatings Preparation

Electroless deposition of NiP and NiP-TNPs composite coatings was carried out on a high strength low alloy (HSLA) steel substrate, which was obtained from Wugang Chang Yu Long Industry and Trade Co., Ltd., ShenZhen, China. The substrate samples have dimensions of 30 × 25 × 2 mm^3^, with an elemental composition, as shown in Table 1.

**Figure 1:**
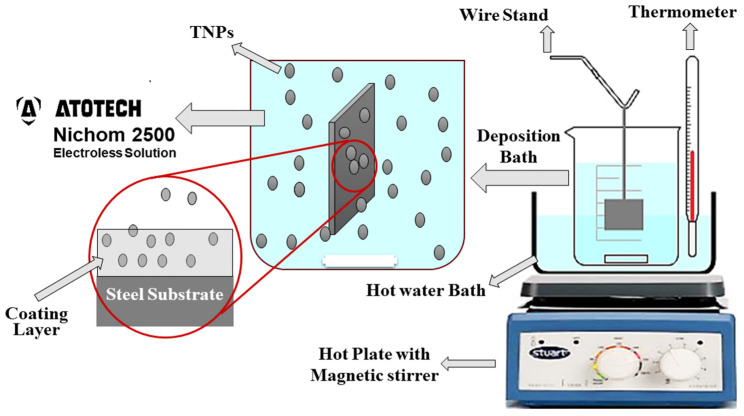
The schematic diagram for the electroless deposition processing.

An analytical grade Titanium powder with 99.9% purity (purchased from US research nanomaterials, Inc, Huston, TX, USA), comprising an average particle size of 70.0 nm, was used as reinforcement material (nanoparticles). Different concentrations 0.25, 0.50, and 1.0 g/L of TNPs were used in electroless deposition (electrolyte) bath for preparation of NiP-TNPs coatings. The electroless deposition solution used in this study was a standard commercial-grade (Nichem 2500 (A and B) from Atotech Inc., Berlin, Germany) solution, with nickel sulfate (Ni_2_SO_4_) as the Ni source, and sodium hypophosphite (NaPO_2_H_2_) as the reducing agent and as a P source. The substrates were ground with different grit SiC abrasive papers (180, 220, 300, 800, 1000, and 2000) successively, to achieve flat and smooth surfaces followed by soap water and ethanol cleaning. The steel substrate samples were washed with deionized water and activated with a 15% solution of H_2_SO_4_ for 30 s. After surface activation, the pretreated specimens were again rinsed with distilled water and immersed directly into the deposition (electroless solution) bath. The preparation of the electroless plating bath was performed by mixing specific quantities of Nichem A and Nichem B, as provided by the manufacturer, in DI water to obtain 1 L of the electroless solution. The temperature of the bath was kept at 86 ± 2 °C for 90 min, which is the deposition time, and the deposition bath was agitated with a magnetic stirrer at 300 rpm to avoid settling of TNPs during deposition processing. For each nanocomposite coating composition preparation, a specific amount of TNPs was added into 1 L of the electroless bath, and the solution was mixed with the ultrasonic probe for 0.5 h, before initiating the deposition process to eliminate any chances of their agglomeration and settling of TNPs during deposition. Then, the cleaned specimens were immersed in the electroless bath, and the deposition process started under the same previous conditions and lasted for 90 min. After that, the coupons were removed, rinsed, and dried. This procedure was repeated for each concentration of TNPs to obtain the different nanocomposite coatings. For simplification, the developed NiP-TNPs nanocomposite coatings were designated as per the TNPs concentrations, which is dispersed into the electroless bath. In this perspective, the obtained coatings samples were labeled as NiP, NiP-0.25TNPs, NiP-0.5TNPs, and NiP-1.0TNPs, respectively. For a clear understanding of electroless deposition processing of NiP-TNPs nanocomposite coatings, a schematic diagram is shown in Figure 1.

### 2.2. Characterization

Various characterization techniques were adopted to characterize and evaluate the performance of developed NiP and NiP-TNPs nanocomposite coatings. The structure and phase analysis of the developed coatings was examined by using the XRD (X-ray diffraction) technique, using a PAN analytical X’pert Pro Cu (Ka), with a scanning rate of 0.02° in the 2θ range from 20° to 80°. Surface morphology and elemental analysis were performed using FESEM (field emission scanning electron microscope) from Nano-450, Netherland, also contain EDX (energy dispersive spectroscopy) analyzer. The topographic study was performed using AFM (atomic force microscope) from Oxford Instruments, Goleta, CA, USA. A coating thickness gage meter (PosiTector 6000) from DeFelsko, Proctor Avenue Ogdensburg, NY, USA was used to measure the thickness of coating samples. The mechanical response of the coatings was studied through microhardness and nanoindentation techniques. Microhardness measurements were performed with Vickers microhardness tester from FM-ARS9000, USA, and nanoindentation measurements were performed using MFP-3D nanoindenter head attached with AFM. For the corrosion study in 3.5 wt. % NaCl solution, electrochemical impedance spectroscopy (EIS) and potentiodynamic polarization (PP) methods were adopted. All electrochemical measurements were performed in an electrochemical cell, having three electrodes systems attached with a GAMRY 3000 potentiostat from Warminster, PA, USA. The calomel electrode was used as a reference, the graphite rod was used as a counter, and coating samples were used as a working electrode. An area of 1 cm^2^ of the coated samples was subjected to the NaCl solution and placed at open circuit potential for 30 min, before starting the electrochemical measurements. For EIS, a 10 mV AC amplitude was used, and the frequency varied from 0.01 to 100 kHz. For PP, a scan rate of 0.167 mV·s^−1^ and a potential range of ±250 mV vs. the open circuit potential was used to acquire the anodic and cathodic polarization curves. To ensure the reproducibility, all tests were repeated three times.

The heat treatment of the developed coatings was commenced in the electric furnace (GSL-1500X-50-UL, MTI Corporation, Richmond, CA, USA). The selected coatings samples were heat-treated at 400 °C for different time intervals (1 h, 10 h, and 50 h) in the open air. Initially, the weight of each sample was measured, followed by heating. After heating at 400 °C, for each time interval, the heat-treated samples were weighed again, and the obtained weights were recorded. For weight measurements, the analytical grade balance (ENTRIS64-1S, Sartorius Lab Instruments, GmbH & Co.KG, Göttingen, Germany) with 0.0001 g sensitivity, was utilized. The coated samples subjected to the high-temperature treatment were analyzed using FESEM, AFM, and XRD techniques to study the surface morphology, topography, and composition of the formed phases.

## 3. Results and Discussion

### 3.1. Morphology of TNPs and Developed Coatings

**Figure 2:**
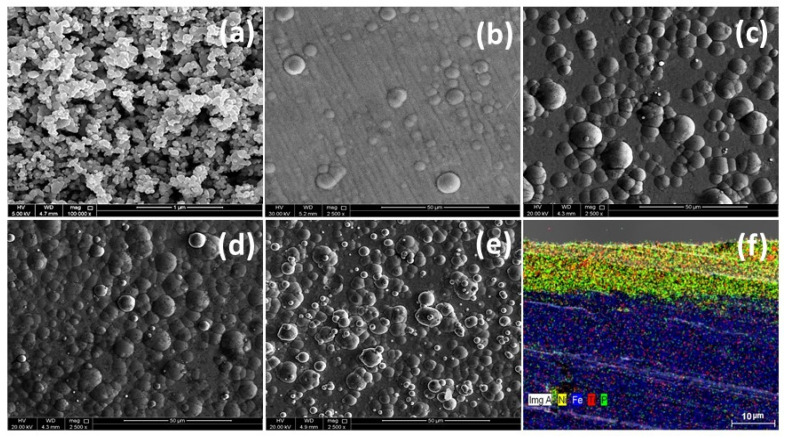
Field emission scanning electron microscope (FESEM) image of the Ti nanoparticles (TNPs)—(**a**), nickel phosphorus (NiP)—(**b**), NiP-0.25TNPs—(**c**), NiP-0.50TNPs—(**d**), NiP-1.0TNPs—(**e**) and cross-section view for NiP-0.50TNPs—(**f**). Elemental mapping of cross-section of NiP-0.50TNPs nanocomposite coatings.

Figure 2a depicts the FESEM image of TNPs showing a spherical morphology with an average particle size of 70 nm, which is consistent with the particular datasheet. The surface morphology of NiP and NiP-TNPs coatings were also studied with FESEM, and outcomes are presented in Figure 2 b–e). Figure 2b corresponds to NiP coatings morphology. It indicates that NiP coatings surface has a smooth nodular structure with some longitudinal lines, which is consistent with the literature [11,12]. FESEM images shown in Figure 2c–e are for NiP-0.25TNPs, NiP-0.5TNPs, and NiP-1.0TNPs coatings, respectively. Comparing these images with the image of NiP coating (Figure 2b), it can be realized that the incorporation of TNPs into the NiP matrix has a substantial effect on the morphology of NiP coatings. The incorporation of TNPs into the NiP matrix resulted in the formation of more nodules, which increases with the increasing concentration of TNPs. This morphology change confirms the effective incorporation of TNPs into the NiP matrix during the deposition process. Furthermore, it is noticed that the size of nodules is reduced as the concentration of TNPs increases. The reduction in globular size can be regarded as the increase in nucleation rate during the deposition process, i.e., the effect of the availability of more nucleation sites during the deposition process. Incorporation of TNPs into the NiP matrix may act as a nucleation site and can accelerate the nucleation rate, which ultimately prevented lateral grain growth and refined the structure. A fine-grained structure can be formed by increasing the number of nucleation sites and decreasing grain growth [14,30]. Further, these images also depict that the globular morphology becomes more homogenous and consistent, while adding more TNPs into the NiP matrix, which eventually seems perfect in NiP-0.5TNPs composite coating (Figure 2d). Moreover, some oversaturation and irregularity in the nodular structure can be seen in Figure 2e. This can be due to the over-concentration of TNPs into the NiP matrix, which may lead to agglomeration of TNPs and finally disturb the structure. The agglomeration of TNPs may have an adverse effect on the mechanical and anticorrosion properties of the developed coatings [31]. Figure 2f is an EDX elemental mapping image of NiP-0.5TNPs nanocomposite coatings that confirms the incorporation of TNPs into NiP matrix uniformly. This image also confirms the good adherence of nanocomposite coatings to the substrate having a thickness of ~11 µm. Moreover, it also depicts that the NiP-0.5TNPs nanocomposite coatings are compact in structure, revealing no defect at the substrate and coating interface.

**Figure 3:**
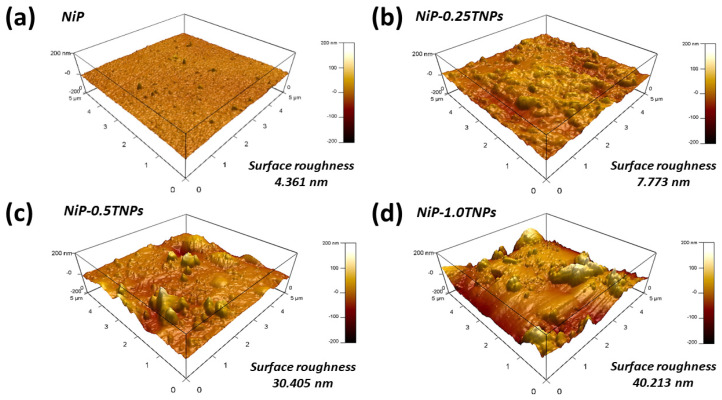
The 3D Surface topographic images for NiP (**a**), NiP-0.25TNPs (**b**), NiP-0.50TNPs (**c**), and NiP-1.0TNPs (**d**) nanocomposite coatings.

Further, to have more details on the surface characteristics of the developed coatings, AFM surface analysis was carried out. The 3D surface profile-images of NiP and NiP-TNPs coatings are shown in Figure 3a–d). From these images, it can be visualized that the addition of TNPs has a pronounced effect on surface profile, topography, and roughness of developed coating. For a clear comparison, the surface roughness parameters for the NiP and NiP-TNPs composite coatings are composed in Table 2. It can be noticed that the average surface roughness of the NiP coating increases with the increasing amount of TNPs (4.3 nm at NiP to 40.2 nm at NiP-1.0TNPs). Moreover, the surfaces of the NiP, NiP-0.25TNPs, and NiP-0.50TNPs coatings consist of peaks and asperities, whereas that of NiP-1.0TNPs coating has a canyon-like structure. The enhancement in surface roughness is because of the increasing amount of TNPs in the nanocomposite coatings. Our results are consistent with previous studies [32,33]. These results suggest that TNPs significantly affect the surface characteristics of the NiP coatings.

### 3.2. Composition Analysis (EDX Scans)

**Figure 4:**
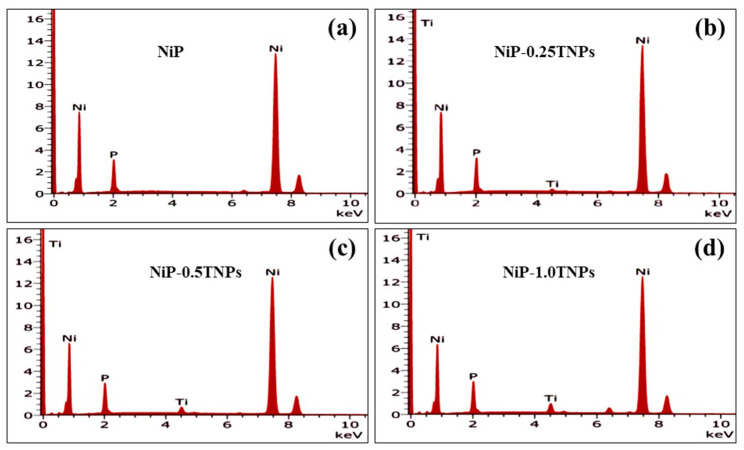
Energy dispersive spectroscopy (EDX) scan spectra of (**a**) NiP, (**b**) NiP-0.25TNPs, (**c**) NiP-0.5TNPs and (**d**) NiP-1.0TNPs nanocomposite coatings.

EDX test was performed to perceive the co-deposition of TNPs into the NiP matrix and to study their effect on the chemical composition of the NiP coatings. Figure 4a–d represents the EDX spectra of NiP, NiP-0.25TNPs, NiP-0.5TNPs, and NiP-1.0TNPs coatings, respectively. From Figure 4a, it can be seen that NiP coating is composed of only Ni (89 wt. %) and P (11.0 wt. %). Whereas, Figure 4b–d indicates the presence of TNPs in the nanocomposite coatings (NiP-TNPs), due to the presence of the “Ti” (along with Ni and P peaks) in the EDX spectra. Furthermore, an increase in TNPs amount into the NiP matrix is also confirmed with an increasing peak intensity of “Ti” in the EDX spectra. This relative increase in TNPs quantity in the obtained coating samples can be correlated with the TNPs concentrations, which were dispersed into the deposition baths as 0.25, 0.5, and 1.0g/L. EDX results can also be used to deduce the actual elemental compositions (wt. %) of each developed coating samples. This actual composition of each coating is presented in Table 3. In short, our EDX analysis confirms the successful incorporation of TNPs in the NiP matrix; these results are consistent with the Balaraju [34] findings, who propose that the quantity of the nanoparticles in the composite coatings depends on the nanoparticles concentration in the deposition bath. 

### 3.3. Structural Analysis (XRD)

**Figure 5:**
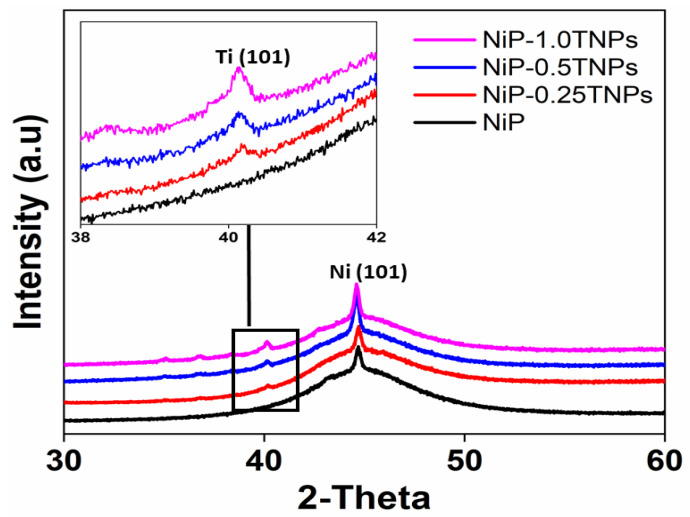
X-ray diffraction (XRD) graphs for NiP and NiP-TNPs nanocomposite coatings.

The phases investigation of the developed coatings was performed through XRD. Figure 5 shows the XRD spectra of NiP-TNPs composite coatings having different content of TNPs. It is seen that a broad peak is formed at 2θ in the range of 40–50° for all coating compositions. The appearance of this broad peak is indicative of the formation of a semi-crystalline structure in the developed coatings. The origin of this structure can be primarily due to the disorder created in the Ni lattice by the incorporation of P (phosphorus) atoms [6]. Moreover, the addition of TNPs into the NiP matrix and the heterogeneous nucleation process during the deposition can also lead to distortion in the lattice structure [6,35,36]. Further, the presence of an additional peak at 2θ~40° in NiP-TNPs composite coatings confirms the successful incorporation of TNPs into the NiP matrix. These findings also agree with our EDX analysis (Figure 4). Furthermore, the increasing intensity of the Ti peak with the increasing amount of TNPs in the coating bath suggests the augmentative trend of Ti co-deposition into the NiP matrix. These findings are also consistent with the literature [29,37].

### 3.4. Mechanical Performance Evaluation

Prior to perform mechanical testing (microhardness and nanoindentation), the thickness of the coating samples was measured using thickness gage meter. Coating thickness measured values for all compositions are presented in Table 4.

#### 3.4.1. Vicker Microhardness Testing

**Figure 6:**
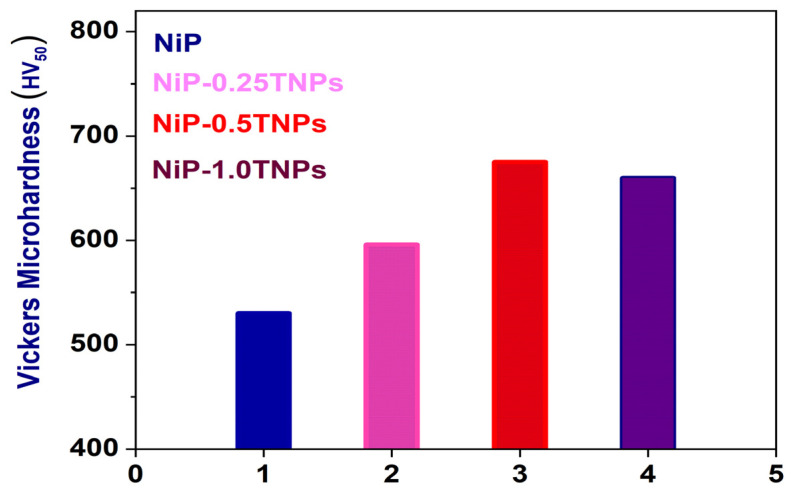
Vickers microhardness results for NiP, NiP-0.25TNPs, NiP-0.5TNPs, and NiP-1.0TNPs nanocomposite coatings.

The microhardness test results of NiP, NiP-0.25TNPs, NiP-0.5TNPs, and NiP-1.0TNPs nanocomposite coatings are presented in Figure 6. It can be observed that all compositions of NiP-TNPs composite coatings demonstrate improved microhardness when compared to the NiP coatings. The NiP-0.5TNPs coating shows the highest microhardness (675 HV_50_) value contributing to ~28% increase in the microhardness when compared to NiP coatings. The increase in the hardness values can be regarded as the effect of dispersion hardening and grain refinement strengthening evolved by the incorporation of TNPs in the NiP matrix. In the dispersion hardening process, it can be assumed that the TNPs can act as an obstacle that does not deform during the deformation, such that the moving dislocations have to bypass the obstacles by changing their deformation path, whereas the grain refinement process can also strengthen the materials by providing obstacles to the dislocation motion at grain boundaries. In our case, TNPs dispersed into the deposition bath may provide various heterogeneous nucleation sites leading to accelerating the nucleation rate, which reduces the lateral growth of the grains and thus ultimately forms a fine structure. This argument can be visualized in FESEM images presented in Figure 2. Moreover, the inclusion of TNPs can also densify the NiP matrix by filling up gaps and by reducing the porosity, thus, leading to improvements in the microhardness [30].

#### 3.4.2. Nanoindentation Testing

**Figure 7:**
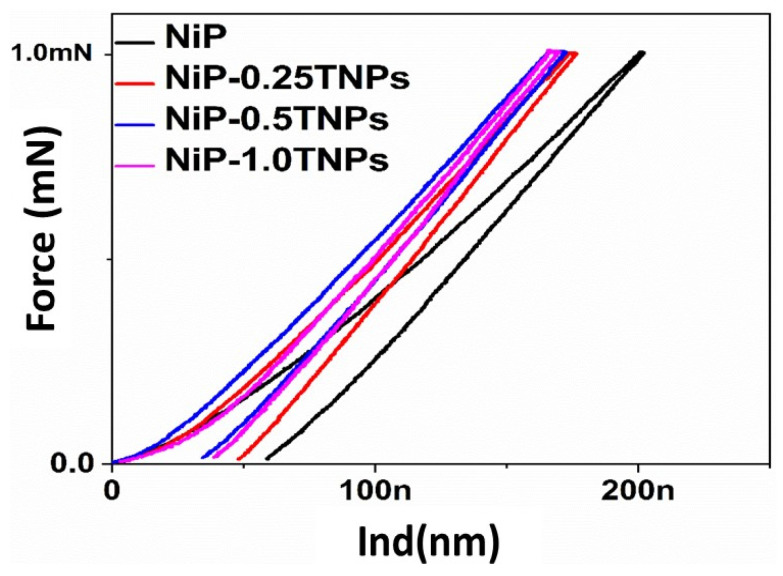
Nanoindentation loading-unloading curve for NiP, NiP-0.25TNPs, NiP-0.5TNPs and NiP-1.0TNPs nanocomposite coatings.

Further, to evaluate the mechanical characteristics of NiP and NiP-TNPs nanocomposites coatings, nanoindentation was performed by using the nanoindenter head (Berkovich diamond indenter tip) connected to AFM, using a maximum 1 mN indentation force at loading and unloading rate of 200 μN/s and having dwell time of 5 s. The load-displacement curves of NiP and NiP-TNPs composite coatings having various concentrations of TNPs are shown in Figure 7. For a clear comparison, the values extracted from the nanoindentation test are also composed in Table 4. For the analysis of nanoindentation results, the Berkovich tip indentation depth and area under the indentation curve are considered more important. From Figure 7, it can be observed that the NiP coatings occupy a more substantial area under the curve than the NiP-TNPs composite coatings. For NiP coatings, the depth of indentation is 58.0 nm, whereas the depth of indentation for NiP-TNPs nanocomposite coatings is smaller than that of NiP coatings. The lowest value of 30.0 nm is shown by the NiP-0.50TNPs composite coating. Reductions in the indentation depth and the area under the curve infer the increase in resistance of the material against deformation. This determines that NiP-TNPs composite coatings are mechanically more robust as compare to the TNPs-free coating. The presence of TNPs in the NiP matrix could act as barriers to retort plastic flow and, thus, eventually lead to an increase in the mechanical strength [30,38,39,40]. Moreover, Figure 7 depicts that all indentation curves are smooth and continuous, which indicates the defect-free fabrication of all coatings. Importantly, a comparison of the shape of nanoindentation curves of NiP coatings with NiP-TNPs nanocomposite coatings suggests that the NiP coatings are relatively brittle as compared to the NiP-TNPs nanocomposite coatings. This is primarily due to the ductile nature of TNPs. These results are consistent with previous reports [41].

### 3.5. Corrosion Resistance Study

The electrochemical impedance spectroscopy (EIS), and potentiodynamic polarization (PP) corrosion measurements were carried out in 3.5 wt. % NaCl solutions at room temperature, to illustrate the corrosion performance of the electroless NiP-TNPs coatings with different Ti nanoparticles concentrations.

#### 3.5.1. Electrochemical Impedance Spectroscopy (EIS)

**Figure 8:**
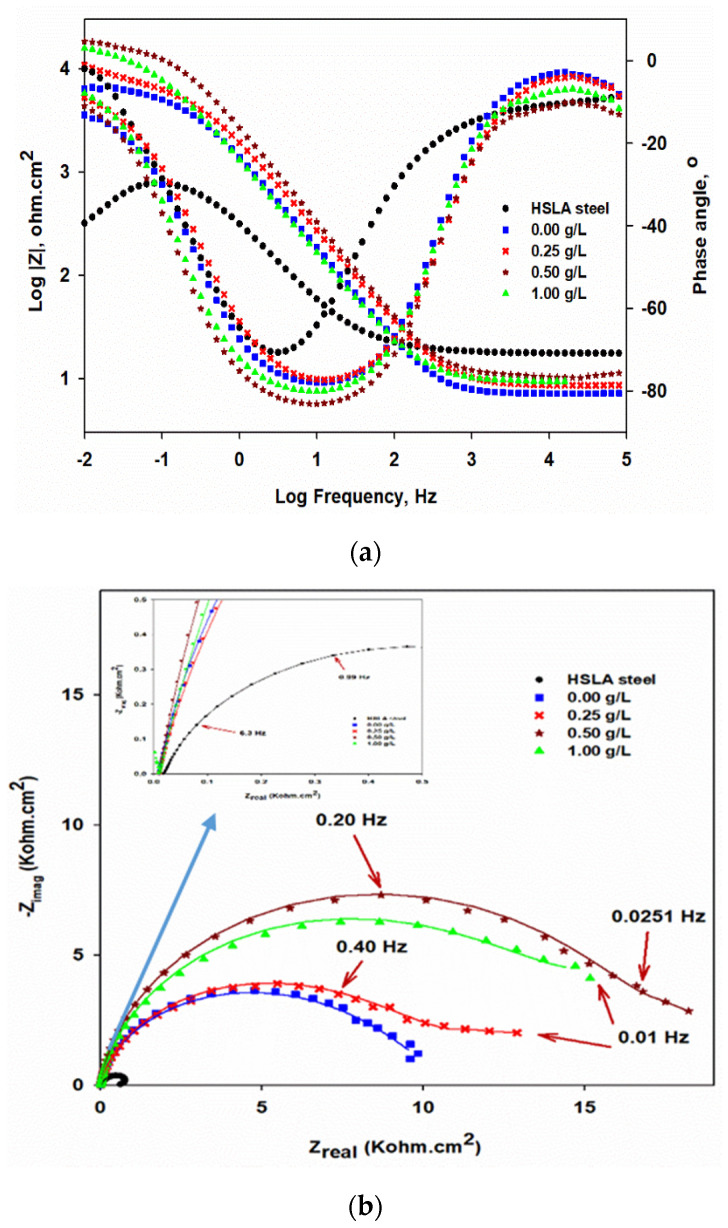
(**a**) Bode-phase angle and (**b**) Nyquist-plots of the HSLA steel, NiP, and NiP-TNPs (NiP-0.25TNPs, NiP-0.50TNPs, and NiP-1.0TNPs) nanocomposite coatings with different content of TNPs that immersed in 3.5 wt. % NaCl solution at 25 °C. The inset in b is the enlargement of the low impedance regions.

**Figure 9:**
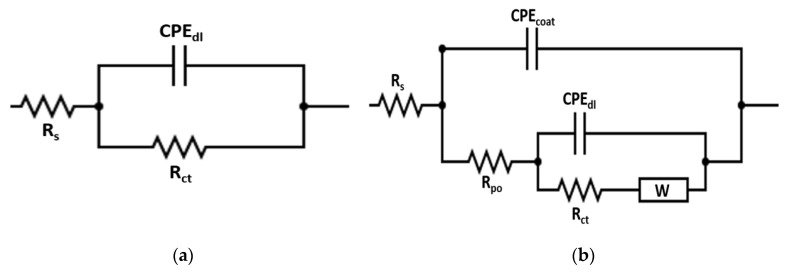
(**a**) One-time constant and (**b**) two–time constants equivalent electric circuits with a diffusion element.

The bode-phase angle plots and the corresponding Nyquist-plots, which are measured at OCP, for the developed coatings are shown in Figure 8a,b, respectively. The inset in Figure 8b is the magnifications of the low impedance regions of the Nyquist plots of the different measured samples. The measured and the fitted EIS data are represented by dots and complete lines, respectively. Figure 9a,b shows the equivalent circuits that were used to fit the EIS data and possessed one and two–time constants with a diffusion component, respectively, depending mainly on the coating type. Different EIS parameters obtained after fitting the measured data are shown in Table 5. The *R*_s_, *R*_po_, and *R*_ct_ are solution, pore, and charge transfer resistances, respectively, CPE_coat_ donates the constants-phase element for the coating and CPE_dl_ is the constant-phase element for the double layer. The low–frequency time constant (CPE_dl_ and R_ct_) demonstrates to the substrate/coating interface, whereas the combination of CPE_coat_ and *R*_po_ represents the high–frequency time constant that corresponds to the coating/solution interface. The *W* is referred to the Warburg diffusion resistance, which clarifies the existence of electrolyte diffusion. The CPE is a pseudo-capacitive element, which is used instead of the regular one due to the surface roughness and the coating inhomogeneity, which is assigned to the irregular thickness of the deposited coatings, in addition to the non-uniform current distribution at the surface [42]. The impedance of CPE is calculated using the following Equation [43];

(1) $$Z_{CPE}\;=\;\frac{1}{Q\;{\left(jw\right)}^{n}}$$

where Q is the CPE constant and equals to the (1/|Z|) at ω = 1 rad/s, j is the imaginary number, ω is the angular frequency of the AC signal (1/rad), and *n* is the CPE exponent. The deviation from ideal behavior is expressed by the exponent *n* (0 ≤ *n* ≤ 1). The ideal resistor is indicated when *n* equals zero, whereas the ideal capacitor is acted when *n* equals 1 [44,45].

As in Figure 8a and Table 5, the impedance value for the HSLA steel substrate is approximately 670 Ω·cm^2^. The impedance value has increased by one order magnitude for electroless NiP coating compared to that of the substrate. The formation of the hypophosphite layer causes the increased impedance value of the NiP coating due to the reaction of the phosphorus with water [46,47]. This layer acts as a protective layer for nickel, since it prevents its further hydration in the corrosive media, resulting in the improvement of the corrosion-protection of the NiP coatings. The impedance of the NiP coating is further increased with the reinforcement of TNPs. As is well known, Ti itself is a corrosion-resistant metal [48], which leads to improving the corrosion-resistance of the NiP coatings. Furthermore, the NiP coatings may be more porous; therefore, it becomes denser with the addition of TNPs, and its porosity decreases, reducing the active sites for the corrosion and preventing the direct contact of electrolyte with the substrate [49]. It can be seen in Table 5, the charge transfer resistances of the NiP-TNPs composite coatings have increased as the concentration of the TNPs in the electroless bath increased to 0.5 g/L, and then it slightly decreased. The highest value of *R*_ct_ is observed at NiP-0.5TNPs (18.6 KΩ·cm^2^), with approximately 96.5% protection efficiency, which is reduced to 15.4 KΩ·cm^2^ at NiP-1.0TNPs, with approximately 95.6% protection efficiency, as shown in Table 5. The slight reduction in the *R*_ct_ value is due to the increase in the content of TNPs, which may generate defects, causing more inhomogeneity in coatings. 

Moreover, it is noticed that the phase-angle plots of the NiP coatings in the absence and presence of TNPs have the same shape and position, as shown in Figure 8a. This indicates that there is a similar fundamental process that takes place on their surfaces. However, they have different peak maximum values, depending on their protection ability. The maximal peak values of the NiP-TNPs coatings are higher than the NiP coatings. As a comparison, the NiP-0.5TNP coatings have the highest peak maximal value, indicating the better corrosion resistance for NiP-TNPs nanocomposite coatings. On the contrary, the phase angle plots of the various developed coatings are different in shape, position, and peak maximum, when compared to that of the substrate. Therefore, the substrate shows one-time constant behavior, whereas the developed coatings display two-time constants fitting, in which two relaxation processes take place. One is due to the coating layer and measured at the intermediate and higher frequencies. The other one is checked at lower rates and related to the electrochemical reactions that happened at the substrate and coating interphase. 

Nyquist plots for HSLA steel, NiP, and NiP-TNPs composite coatings with different content of TNPs are depicted in Figure 8b. The semicircle radius of Nyquist curves significantly increased for NiP and NiP-TNPs composite coatings as compared to HSLA steel, the increase in impedance values for Nyquist plots are also consistent with their Bode plots. 

Furthermore, the decrease in porosity of the NiP coating with the addition of TNPs also helps to increase the pore resistance (*R*_po_) of the NiP-TNPs coatings. The values of *R*_po_ are given in Table 5. The pore resistance (*R*_po_) of NiP-TNPs coatings increases with the addition of TNPs and reaches its highest value at NiP-0.5TNPs composition. Furthermore, the decrease in the amount of constant phase element for NiP-TNPs nanocomposites coatings as compared to HSLA steel and NiP coating proves their better corrosion-resistant behavior. It is worth mentioning that the inhibition efficiency (*I.E*) of the coatings was calculated by using the following equation [47].

*I.E* (%) = (1 − *R*_ct2_/*R*_ct1_) × 100 (2)

where the *R*_ct2_ and *R*_ct1_ are the charge-transfer resistance of the substrate and coating, respectively. The inhibition efficiency for NiP and NiP-TNPs is given in Table 5. 

#### 3.5.2. Potentiodynamic Polarization Technique

**Figure 10:**
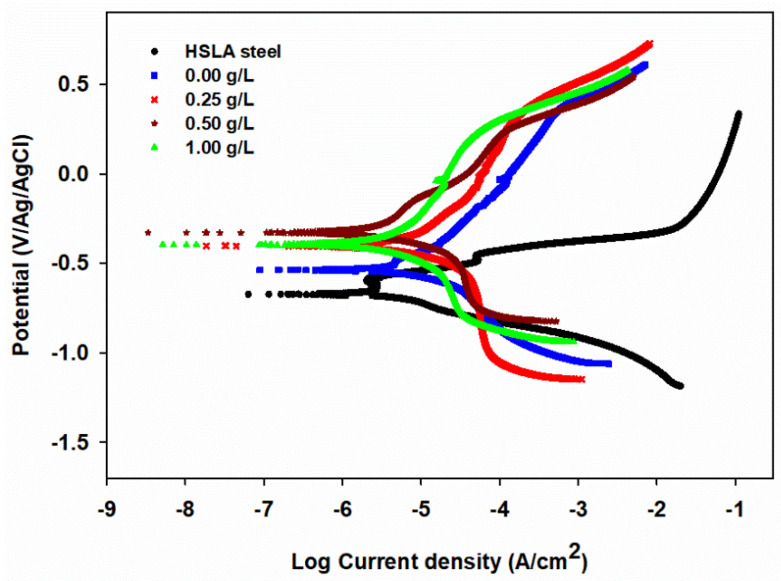
Tafel curves for HSLA steel, NiP, and NiP-TNPs nanocomposite coatings (NiP-0.25TNPs, NiP-0.5TNPs, and NiP-1.0TNPs) immersed in 3.5 wt. % NaCl solution at 25 °C and a scan rate of 0.167 mV·s^–1^.

Figure 10 shows the Tafel plots (Potentiodynamic polarization curves) of HSLA steel, NiP, and NiP-TNPs nanocomposite coatings (NiP-0.25TNPs, NiP-0.5TNPs, and NiP-1.0TNPs) that are immersed in 3.5 wt. % NaCl at 25 °C. Table 6 summarizes the various electrochemical parameters: *i*_corr_ (corrosion-current density), *E*_corr_ (corrosion-potential), *b*_c_ and *b*_a_ (anodic and cathodic Tafel slopes) that acquired using the Tafel extrapolation method, and the corrosion inhibition efficiency (*IE*%) for all coatings. The inhibition efficiency (*IE*%) for the coated samples derived from the data obtained from Tafel plots is obtained by applying the following equation [47].

(*IE* %) = (1 − *i*_corr2_/*i*_corr1_) × 100 (3)

where the *i*_corr1_ and *i*_corr2 are_ the corrosion current density of the substrate and coating samples, respectively. The corrosion-potential (*E*_corr_) for HSLA steel was around −764 mV, while that for the NiP coating is shifted toward more positive value (−527 mV), which is further anodically shifted to −383, −311, and −388 mV by the addition of TNPs (amounting 0.25, 0.5, and 1.0 g/L, in electroless deposition bath correspondingly) in the NiP matrix. The reduction in the corrosion potential of the NiP-TNPs nanocomposite coatings compared to NiP coatings proves their better corrosion resistance. The more negative value of the corrosion potential of HSLA steel indicates that the steel’s surface is more susceptible to corrosion. Moreover, a noteworthy decrease in the corrosion-current density values of the coated samples compared to the values for the substrate is observed, such that the icorr value is dropped from 69 µA·cm^−2^, in the case of the substrate, to 8.2 µA·cm^−2^ for NiP coating. A further drop in the icorr value to 6.5 µA·cm^−2^ takes place at NiP-0.25TNPs coating, signifying an increase in the corrosion resistance of the NiP coating in the presence of TNPs nanoparticles. As the concentration of the TNPs particles is increased into the NiP matrix, the icorr of the NiP-TNPs nanocomposite coating decreases and reaches to the highest drop at NiP-0.5TNPs composite coating composition, as shown in Figure 10 and Table 6. Further increase in the TNPs concentration into NiP matrix (NiP-1.0TNPs nanocomposite coating), led to an increase of about 27.5% in icorr of the NiP-1.0TNPs coating, resulting in a reduction in its protection efficiency of about 1.6%, compared to that of the NiP-0.5TNPs nanocomposite coating. Distinctly, the NiP-0.5TNPs nanocomposite coating has the highest corrosion protection efficiency, reaching 94.2%, as shown in Table 6. The change in the values of anodic and cathodic Tafel slopes (*b*_a_, *b*_c_) indicates the change in the mechanism of iron dissolution due to the deposition process. The results of EIS and potentiodynamic polarization measurements are consistent and confirmed with each other.

### 3.6. Heat Treatment Analysis

#### 3.6.1. Surface Morphology

**Figure 11:**
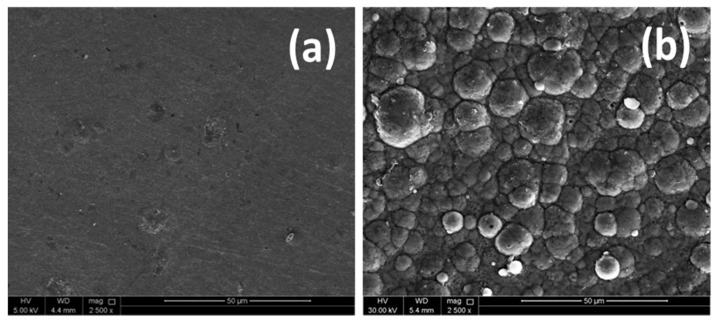
SEM micrographs of (**a**) NiP and (**b**) NiP-0.5TNPs composite coatings after heat treatment for 50 h in air.

For the heat treatment study, NiP, NiP-0.5TNPs nanocomposite coating samples were selected and heat-treated at 400 °C in an open furnace, for different time intervals (1.0 h, 10.0 h, and 50.0 h). Figure 11a,b depicts the surface morphology of the heat-treated coated samples after the 50.0 h heat-treatment process. A comparison of the corresponding SEM images of non-heat-treated coated samples Figure 2b,d indicates that the nodular structure of NiP coatings is slightly modified. However, the globular structure of NiP-0.5TNPs coatings remains unchanged without any noticeable grain coarsening, suggesting that the incorporation of TNPs into the NiP matrix has made the structure more stable against the oxidation. The consistency in the grain structure is useful in maintaining the surface and structural properties of the coatings. Moreover, no cracks, voids, and peeling off the coatings from the substrate were noticed, demonstrating moderate performance in heat treatment processing (oxidizing) and adhesion along with the substrate showing the same thermal expansion behavior. 

**Figure 12:**
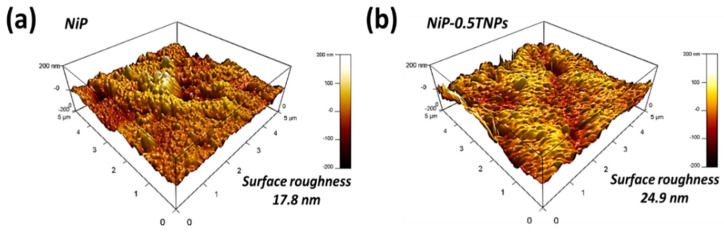
The 3D Surface topographic images for NiP (**a**) and NiP-0.5TNPs (**b**) coatings of 50 h heat treatment in an open furnace condition.

AFM was also performed on heat-treated samples to quantify the surface characteristics. The 3D surface topographic images of NiP and NiP-0.5TNPs, coatings are shown in Figure 12. The surface-roughness parameters and their corresponding values are summarized in Table 7. Comparing these results with the corresponding values of as developed coatings (as shown in Table 2), it can be perceived that the surface roughness for the NiP samples is increased after the heat treatment process, whereas the surface roughness for the NiP-0.5TNPs nanocomposite coating samples has decreased. The decrease in surface roughness might be due to the diffusion of TNPs and Ni atoms on the surface area. In contrast to volume diffusion, surface diffusion is relatively easier, because of the presence of smaller constraints on the diffusion of atoms towards the surface [50]. It can also help to fill the pits of the specimen on the surface with TNPs and Ni atoms. This indicates the structural stability and heat resistance of the NiP-0.5TNPs nanocomposite coatings. The inherent high surface roughness of NiP-0.5TNPs nanocomposite coatings is obviously due to the presence of TNPs [51].

#### 3.6.2. Phase and Composition Analysis after Heat Treatment

**Figure 13:**
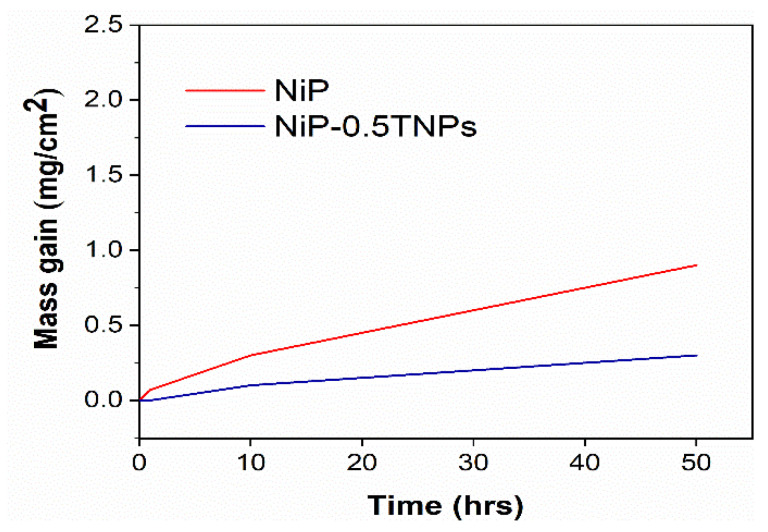
Mass gain vs. time graph for NiP and NiP-0.5TNPs coatings heat-treated at 400 °C in air.

**Figure 14:**
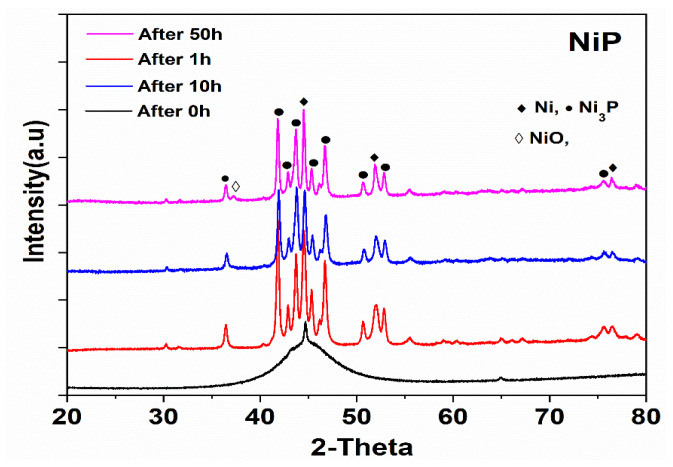
XRD spectra of NiP coatings after heat treatment in air at different time intervals.

**Figure 15:**
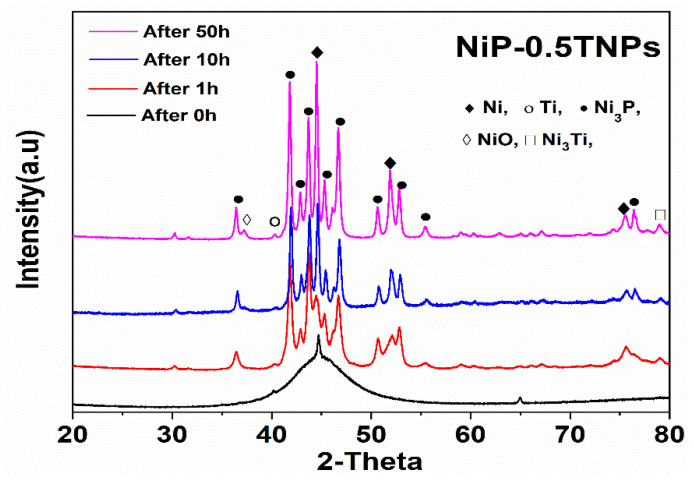
XRD spectra of NiP-0.5TNPs coatings after heat treatment in air at different time intervals.

Figure 13 presents the weight gain during open furnace heat treatment of NiP and NiP-0.5TNPs composite coatings. These curves were obtained after heating (oxidizing) the coatings at 400 °C for different time intervals (1.0, 10.0, 50.0 h). It can be noticed that NiP-0.5TNPs composite coatings show a negligible mass gain indicating better thermal resistance compared with the NiP coating. This improvement in thermal stability can be ascribed to the presence of significant contents of TNPs in the NiP matrix, which is itself thermally more stable [52]. The dispersion of the TNPs into the NiP matrix also restricts the diffusion of oxygen atoms, resulting in slowing down the outward to inward atomic diffusion [53]. Similarly, various phases formed during the subsequent heating treatment process were identified using the XRD technique. In the case of NiP coatings, the developed structure consists of Ni, NiO, and Ni_3_P phases (Figure 14), whereas the NiP-0.5TNPs composite coatings are composed of Ni, NiO, Ni_3_P, Ni_3_Ti (Figure 15). The presence of Ti and Ni_3_Ti peaks in the NiP-0.5TNPs coatings can be ascribed to the presence of TNPs in the NiP matrix. The TNPs present in the NiP matrix defuse into the NiP matrix and restrict the atomic diffusion. This mechanism makes the structure more stable and heat resistant. To summarize, the superior and durable oxidizing resistance performance of NiP-0.5TNPs composite coatings is associated with the incorporation of TNPs in the NiP matrix and the formation of more stable and thermally resistant phases, such as NiO, Ni_3_P, Ni_3_Ti [54,55].

## 4. Conclusions

NiP-Ti nanocomposite (NiP-TNPs) coatings containing various content of TNPs were successfully deposited on HSLA steel through the electroless deposition technique. The FESEM and EDX analysis confirmed the progressive co-deposition of TNPs into NiP matrix, whereas phase pure structure formation was perceived by X-ray analysis. Microhardness and nanoindentation results report the significant influence of TNPs on the mechanical properties of NiP coatings that reached to its ultimate values for the NiP-0.5TNPs nanocomposite coating composition. Similarly, the EIS results confirm the excellent corrosion inhibition efficiency of the NiP coatings with an increasing amount of TNPs, reaching to ~96.4% with NiP-0.5TNPs composite coating composition. Moreover, the heat resistance (oxidizing resistance) behavior of the NiP coating was remarkably increased, due to the presence of TNPs into the NiP matrix and the formation of more stable (heat resistant) phases, such as Ni_3_P, Ni_3_Ti, NiO, etc., during the subsequent processing. Briefly, the NiP-0.5TNPs composite coatings prepared from 0.5 g/L TNPs concentration in electroless deposition bath, demonstrate superior mechanical, exceptional anticorrosion, and decent heat resistant characteristics and, thus, suggesting them an attractive coatings material.

## Figures and Tables

**Table 1:** The elemental composition of the high strength low alloy (HSLA) steel sheet.

Element	C	Si	Mn	Ni	Cr	S & P	Balance
**wt. %**	0.28	1.2	0.9	0.3	1.1	0.025	Fe

**Table 2:** Comparison of roughness parameters for NiP, NiP-0.25TNPs, NiP-0.5TNPs and NiP-1.0TNPs nanocomposite coatings.

	Parameters	Measured Values			
1	Composition	NiP	NiP-0.25TNPs	NiP-0.5TNPs	NiP-1.0TNPs
2	Average roughness (R_ave_.), nm	4.3	7.7	30.4	40.2
3	Root Mean Square roughness (R_RMS_), nm	5.6	11.3	42.8	58.5

**Table 3:** Elemental compositions of the different obtained coatings.

Sr.No	Sample Designation	Ni (wt. %)	P (wt. %)	Ti (wt. %)
1	NiP	89.15	10.85	0.0
2	NiP-0.25TNPs	88.81	10.60	0.6
3	NiP-0.5TNPs	88.05	10.10	1.85
4	NiP-1.0TNPs	86.77	10.01	3.22

**Table 4:** Coating thickness and nanoindentation mechanical results for NiP, NiP-0.25TNPs, NiP-0.50TNPs and NiP-1.0TNPs nanocomposite coatings.

	Parameter	Measured Values			
1	Composition	NiP	NiP-0.25TNPs	NiP-0.5TNPs	NiP-1.0TNPs
2	Coating Thickness (µm)	12	12	11	10
3	Modulus(E) GPa	12.88	16.21	18.26	17.48
4	Stiffness, kN/m	7.52	8.5	9.02	8.22

**Table 5:** Electrochemical impedance spectroscopy (EIS) fitting results of the HSLA steel NiP and NiP-TNPs nanocomposites coatings that immersed in 3.5 wt. % NaCl solution at 25 °C.

Composition	*R*_s_ (Ω·cm^2^)	*R*_po_ (Ω·cm^2^)	CPE_coat_ × 10^−6^ (S^n^ Ω^−1^·cm^−2^)	m	*W* (S_s^1/2^)	*R*_ct_ (Ω·cm^2^)	CPE_dl_ × 10^−6^ (S^n^ Ω^−1^·cm^−2^)	*n*	*IE* (%)
HSLA steel		---	---		---	670	450	---	00.0
NiP		500	59.9	0.40	2.698 × 10^−3^	8800	74.3	0.93	92.4
NiP-0.25TNPs		1300	56.0	0.30	3.034 × 10^−3^	11600	67.0	0.90	94.2
NiP-0.50TNPs		2100	24.8	0.80	1.712 × 10^−3^	18600	63.6	0.95	96.5
NiP-1.0TNPs		1900	43.0	0.90	1.298 × 10^−3^	15440	65.0	0.90	95.6

**Table 6:** Tafel fitting results of the HSLA steel, NiP, and NiP-TNPs composite coatings (NiP-0.25TNPs, NiP-0.5TNPs, and NiP-1.0TNPs) that immersed in 3.5 wt. % NaCl solution at 25 °C.

Composition	*−E*_corr_ (mV)	*i*_corr_ (µA·cm^−2^)	*b*_a_ (V/decade)	*b*_c_ (V/decade)	*IE* (%)
HSLA steel	674	69	0.79	0.52	---
NiP	527	8.2	0.48	0.22	88.1
NiP-0.25TNPs	383	6.5	0.29	0.14	90.5
NiP-0.5TNPs	311	4.0	0.14	0.11	94.2
NiP-1.0TNPs	388	5.1	0.16	0.10	92.6

**Table 7:** Comparison of the roughness parameters for NiP and NiP-0.5TNPs nanocomposite coating after 50 h heat treatment in an open furnace condition.

	Parameters	Measured Values	
1	Composition	NiP	NiP-0.5TNPs
2	Average roughness (Rave.), nm	17.8	24.9
3	Root Mean Square roughness _(RRMS)_ nm	24.3	32.05
